# Manipulating exciton dynamics of thermally activated delayed fluorescence materials for tuning two-photon nanotheranostics†

**DOI:** 10.1039/c9sc05817f

**Published:** 2019-12-11

**Authors:** Ya-Fang Xiao, Jia-Xiong Chen, Shengliang Li, Wen-Wen Tao, Shuang Tian, Kai Wang, Xiao Cui, Zhongming Huang, Xiao-Hong Zhang, Chun-Sing Lee

**Affiliations:** Center of Super-Diamond and Advanced Films (COSDAF), Department of Chemistry, City University of Hong Kong Hong Kong SAR P. R. China lishengliang@iccas.ac.cn apcslee@cityu.edu.hk; Jiangsu Key Laboratory for Carbon-Based Functional Materials & Devices, Institute of Functional Nano & Soft Materials (FUNSOM), Joint International Research Laboratory of Carbon-Based Functional Materials and Devices, Soochow University 199 Ren'ai Road Suzhou 215123 Jiangsu P. R. China xiaohong_zhang@suda.edu.cn

## Abstract

Rational manipulation of energy utilization from excited-state radiation of theranostic agents with a donor–acceptor structure is relatively unexplored. Herein, we present an effective strategy to tune the exciton dynamics of radiative excited state decay for augmenting two-photon nanotheranostics. As a proof of concept, two thermally activated delayed fluorescence (TADF) molecules with different electron-donating segments are engineered, which possess donor–acceptor structures and strong emissions in the deep-red region with aggregation-induced emission characteristics. Molecular simulations demonstrate that change of the electron-donating sections could effectively regulate the singlet–triplet energy gap and oscillator strength, which promises efficient energy flow. A two-photon laser with great permeability is used to excite TADF NPs to perform as theranostic agents with singlet oxygen generation and fluorescence imaging. These unique performances enable the proposed TADF emitters to exhibit tailored balances between two-photon singlet oxygen generation and fluorescence emission. This result demonstrates that TADF emitters can be rationally designed as superior candidates for nanotheranostic agents by the custom controlling exciton dynamics.

## Abstract 2

Exciton dynamics can be manipulated rationally in the design of TADF materials for nanotheranostics. Regulating the Δ*E*_ST_ and *f* promises efficient energy flow for tailoring balances between singlet oxygen generation and fluorescence emission.

## Graphical abstract

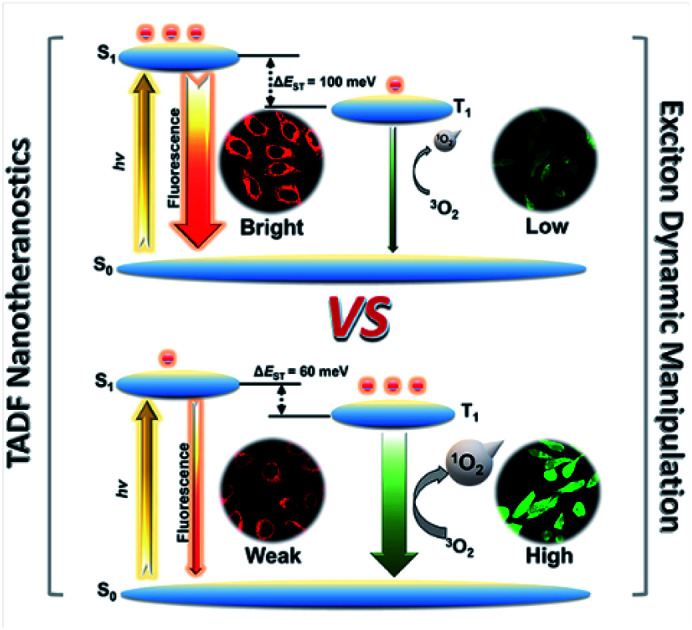

## 1. Introduction

Photodynamic therapy (PDT), with high spatiotemporal resolution and minimal invasiveness, has been considered as a promising cancer therapeutic platform.^1–4^ For PDT using an organic photosensitizer (PS), energy transfer processes occur in sequence: first, a photon transfers its energy to the PS which then forms a singlet (S_1_) excited state; then, the S_1_ state transforms to the lowest triplet (T_1_) state through an intersystem crossing (ISC) process; finally, the T_1_ state decays back to the ground state by passing its energy to the surrounding oxygen leading to the formation of reactive singlet oxygen (^1^O_2_) or by transferring an electron to cellular substrates forming other toxic reactive oxygen species (ROS) such as superoxide anion free radicals and hydroxyl free radicals.^5,6^ The ROS can then destroy various biomolecules (*e.g.* protein and nucleic acid) and kill cancer cells.^7^ Unfortunately, due to the notorious aggregation-caused quenching (ACQ) effect, conventional PSs generally suffer from low-efficient ROS generation.^8,9^ To improve therapeutic effectiveness, many approaches have been proposed to increase the efficiency of ROS production by increasing the rate of the ISC process and overcoming the ACQ effect.^10,11^ So far, adding heavy atoms (*e.g.* halogen and heavy-metal atoms) into the chromophores of the PSs is one of the most widely used strategies. The heavy atoms can significantly enhance spin–orbit coupling in the PS molecules and thus enhance the rate of the ISC process for improving PDT efficacy.^12,13^ However, the heavy atoms can induce intrinsic cytotoxicity limiting their potential clinical applications.^14–16^ Therefore, it is desirable to explore the design principles of highly efficient PSs without using heavy atoms.

Exciton dynamics play a crucial role in the energy disposition of light-harvesting materials, and the energy-flow process can further determine the partition coefficient between the S_1_ state and the T_1_ state. Recently, thermally activated delayed fluorescence (TADF) organic materials with tunable Δ*E*_ST_ and oscillator strength (*f*) have witnessed tremendous development in the organic light-emitting diode (OLED) field.^17,18^ By changing the electron donor (D) and the acceptor (A) segments of TADF molecules, overlaps between their highest occupied molecular orbital (HOMO) and lowest unoccupied molecular orbital (LUMO) can be custom-tuned to obtain tailored Δ*E*_ST_ and *f*.^19^ Upon irradiation, the S_1_ excitons may directly decay by emitting light or convert into T_1_ excitons *via* the ISC process.^20–22^ For a molecule with smaller Δ*E*_ST_ and *f*, the S_1_ excitons tend to transfer into the T_1_ state. As mentioned, the T_1_ state can pass energy to the surrounding oxygen leading to ROS generation for PDT.^23,24^ On the other hand, for a molecule with larger Δ*E*_ST_ and *f*, most of the S_1_ excitons tend to decay back to the ground state favoring fluorescent emission instead of ROS generation.^25^ These suggest that by tuning the electronic structures of the TADF molecules, it is possible to obtain materials with tunable photochemical properties tailored for different biomedical applications from PDT to fluorescence imaging. However, the exploration of TADF nanotheranostic materials is still in its infancy.

Photosensitizers with two-photon absorption play an important role in bio-applications. The excitation wavelength range of most commercial photosensitizers is located in the visible light section (400–700 nm). However, in a biological environment, the wavelength of light from 400 nm to 700 nm could be mainly limited by the shallow penetration depth of tissues.^26^ Near-infrared (NIR) irradiated PSs (excitation wavelength: 700–1000 nm) could relieve such a problem mentioned above, but they are very hard to design because the triplet electronic energy level of the PSs must be higher than the singlet energy level of the oxygen molecule.^27^ To tackle these dilemmas, two-photon excited photosensitizers are proposed to fulfill the two-photon theranostics.^28^

**Scheme 1:**
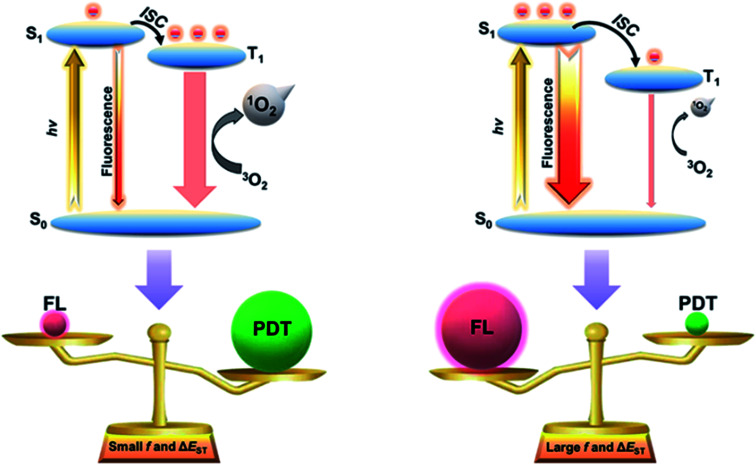
Exciton dynamics in TADF molecules. FL: fluorescence.

In this work, we report guidelines for designing photosensitizers and fluorescent probes with high-efficient reactive oxygen sensitization and intensive fluorescence emission by manipulating Δ*E*_ST_ and *f*. Two TADF molecules with aggregation-induced emission (AIE) characteristics, named PT and AT, have been rationally designed and synthesized. Due to the different electron-donating strengths of the D segments, PT and AT realize tunable Δ*E*_ST_ and *f*, leading to the control of excitons for ROS generation and fluorescent emission (Scheme 1). Small Δ*E*_ST_ and *f* are favorable for PDT due to a more efficient ISC process. In contrast, large Δ*E*_ST_ and *f* are found to be beneficial for fluorescence imaging. The manipulation of Δ*E*_ST_ and *f* are unprecedentedly exploited as a rational strategy for developing high-performance nanotheranostic candidates, and expand the application range of TADF materials.

## 2. Results and discussion

### 2.1 Synthesis and characterization of TADF molecules

Synthetic routes and procedures of PT and AT are shown in Scheme S1 and the Experimental section in the ESI,† respectively. The intermediate products of aromatic amines are first synthesized from the corresponding bromide and 2-chloroaniline. Then the resulting aromatic amines are self-cyclized to form 13,13-dimethyl-5-phenyl-11,13-dihydro-5*H*-indolo[2,3-*b*]acridine (A) and 12-phenyl-5,12-dihydroindolo[3,2-*b*]phenoxazine (P). Finally, Buchwald–Hartwig cross-coupling reaction is carried out between A or P and 3-bromothianthrene 5,5,10,10-tetraoxide (T) to obtain the TADF molecules. Structures of all synthesized compounds have been confirmed with mass spectrometry (MS) and nuclear magnetic resonance (NMR) shown in the ESI (Fig. S1–S6†).

**Fig. 1:**
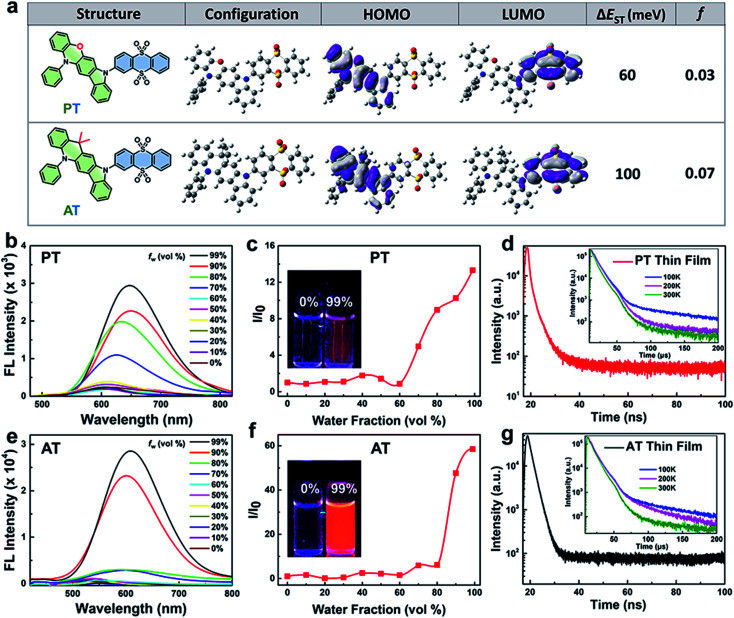
Molecular dynamic simulations and optical characteristics. (a) Molecular structures, configuration and the calculated spatial distributions of the HOMO and LUMO and the value of Δ*E*_ST_ and *f* of AT and PT. Fluorescence spectra of (b) PT and (e) AT in THF/water mixtures with different water fractions (0–99%). Plot of the relative FL intensity (*I*/*I*_0_) of (c) PT and (f) AT with different water fractions (*I*_0_ refers to fluorescence intensity when the *f*_w_ is 0%). Inset photo: fluorescence images of TADF NPs in pure THF solution and 99% *f*_w_ of mixtures under 365 nm irradiation. Prompt transient PL decay curves at room temperature and delayed transient PL decay curves at different temperatures (inset figures) of (d) PT and (g) AT thin films.

To better understand the exciton dynamics, density functional theory (DFT) calculations were then carried out. As shown in Fig. 1a, due to the strong electron-donating nature of phenoxazine, the HOMO in PT is mainly located on the P moiety, whereas the LUMO is mostly distributed over the electron-withdrawing T unit. Thus, the conjugation between P and T is extremely weak, leading to a small Δ*E*_ST_ of 60 meV which is beneficial for the ISC process. While for AT, the weaker electron-donating strength of 9,9-dimethyl-9,10-dihydroacridine makes the HOMO distribution extend to the T segment, leading to more conjugation between A and T. This increases the Δ*E*_ST_ value to 100 meV, resulting in a less efficient ISC process. However, the *f* value of AT is significantly improved to 0.07 compared to 0.03 of PT, which is beneficial for radiative transition. These results reveal that the enhancement of the electron-donating ability of the D segment can decrease the Δ*E*_ST_ value when they connect to the same A unit, which can accelerate the rate of ISC. On the other hand, the increasing overlap between HOMO and LUMO may increase the *f* value, and thus be beneficial for fluorescent emission.^29^ Interestingly, adding water to the THF solution dramatically enhanced the emission of the two molecules, and when the water fraction (*f*_w_%) in the mixed solvent increased to 99%, obvious broad and structureless emissions were observed, and the fluorescence intensity of PT and AT increased by about 13 and 58 times compared to their THF solution, respectively (Fig. 1b, c, e and f), indicating charge transfer (CT) and AIE characteristics. The photoluminescence quantum yields (PLQYs) of PT and AT in a mixed solvent containing over 99% water are measured to be 2.2% and 9.1%, respectively and in the corresponding neat thin films, PT and AT exhibit PLQYs as high as 7.9% and 17%, respectively. The AIE feature of PT and AT is conducive to reduce the non-radiated pathway by the restriction of molecular rotations and vibrations and increase the utilization of the absorbed photons for radiative transition.^30^ Also, as shown in Fig. S7,† decreasing the temperature for both emitters in 2-MeTHF increases the fluorescence and blue-shifts the emissions, which further confirms that the two molecules we proposed are not typical TICT emitters.^31–33^ To confirm their TADF properties, transient PL decays are measured in a nitrogen atmosphere. As shown in Fig. 1d and g, both neat thin films of AT and PT show prompt decays in the range of 100 ns and delayed profiles in the range of 200 μs at room temperature, which are similar to most TADF emitters.^19,34,35^ More importantly, the delayed lifetimes of both thin films decrease along with the increase of temperature from 100 K to 300 K, revealing that the rate of reverse ISC from T_1_ states to S_1_ states increases with the rising temperature,^22^ which further confirm that AT and PT are TADF molecules.

### 2.2 Preparation, characterization and cellular imaging of TADF nanoparticles

**Fig. 2:**
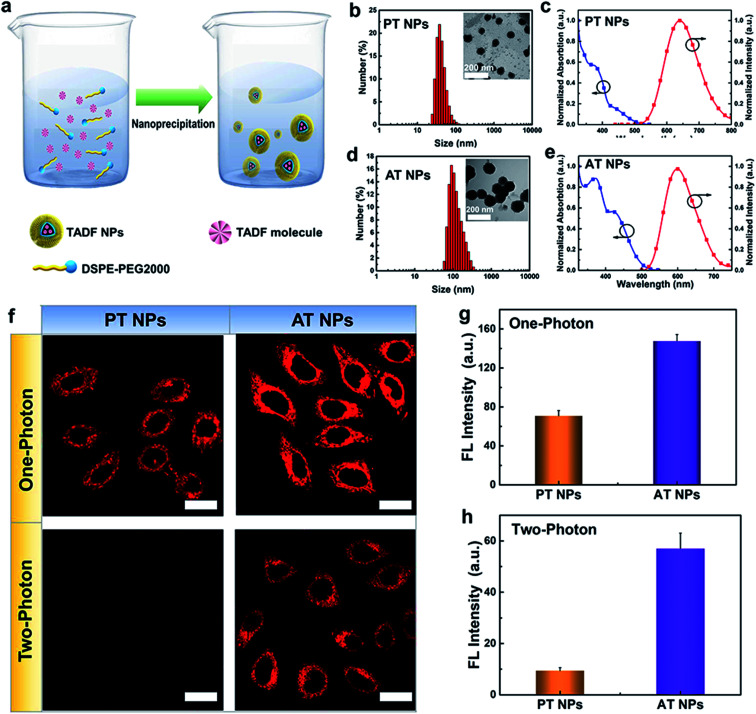
Nanoparticle preparation and cell imaging. (a) Schematic illustration of the nanoprecipitation for nanoparticle preparation. Size distribution and TEM images (inset) of (b) PT NPs and (d) AT NPs. Absorption and fluorescence spectra of (c) PT NPs and (e) AT NPs. (f) Fluorescence images of TADF NPs in HeLa cells irradiated using a xenon lamp (one-photon light resource) and femtosecond laser (two-photon laser, 800 nm). Fluorescence intensities of AT NPs and PT NPs induced by (g) one-photon irradiation and (h) two-photon irradiation from (f).

To enhance the stability and biocompatibility of PT and AT in an aqueous environment, an amphiphilic block copolymer DSPE-PEG 2000 was used to encapsulate AT and PT molecules to form nanoparticles as PT NPs and AT NPs (Fig. 2a). Size distributions of PT and AT NPs measured by DLS are mainly ∼40 nm (Fig. 2b) and ∼100 nm (Fig. 2d), respectively. PT NPs and AT NPs have absorption mainly around 300–400 nm and the peaks of emissions at deep red and red regions of 650 nm and 600 nm, respectively (Fig. 2c and e). Besides, we also confirm that both AT NPs and PT NPs display double decay components in the fluorescence lifetime, which is similar to neat films (Fig. S8†).

Next, we explore the feasibility of exciting the two TADF NPs using NIR radiation *via* a two-photon laser. Two-photon excited (TPE) emission spectra were measured using a fluorospectrophotometer with a femtosecond pulse laser. As shown in Fig. S9 and S10,† both AT NPs and PT NPs display TPE emission excited by a two-photon laser from 760 nm to 800 nm, indicating their potential as two-photon absorption agents. Impressively, AT NPs show stronger emission intensity than PT NPs under the same conditions, revealing that more S_1_ excitons of AT NPs decay back to the ground state. Cell imaging capability of TADF NPs induced by two-photon irradiation was investigated using the HeLa cell line and confirmed by one-photon irradiation as well. As shown in Fig. 2f, both one-photon and two-photon images of AT NPs show a bright red fluorescence signal inside cells, while PT NPs exhibit a weaker fluorescence signal in the one-photon channel and hardly have any signals in the two-photon channel. The two-photon and one-photon induced fluorescence intensities of AT NPs are 6.3 and 2.0 times higher than that of PT NPs, respectively (Fig. 2g and h), which correspond to our previous theoretical calculations that larger Δ*E*_ST_ and *f* of AT will be beneficial for the fluorescence emission.

Distributions of AT NPs and PT NPs in HeLa cells were investigated by staining nuclei with a nuclear-specific dye Hoechst 33342 (blue fluorescence) and the result indicates that they do not enter nuclei and mainly distribute in the cytoplasm area after internalization (Fig. S11†). To further study the ability of colocalization of TADF NPs with other organelles, we co-stained HeLa cells with the TADF NPs and a mitochondria-specific dye MitoTracker green or a lysosome-specific dye LysoTracker green. As shown in Fig. S12,† it is found that TADF NPs have a higher Pearson's colocalization coefficient (∼0.7) with mitochondria than that (∼0.5) of lysosomes, indicating a better targeting ability for mitochondria. Thus, these mitochondria-located TADF NPs can availably destroy the important metabolic organelles with singlet oxygen, leading to more efficient cytotoxicity than non-specific PSs.

### 2.3 Singlet oxygen generation of TADF nanoparticles

**Fig. 3:**
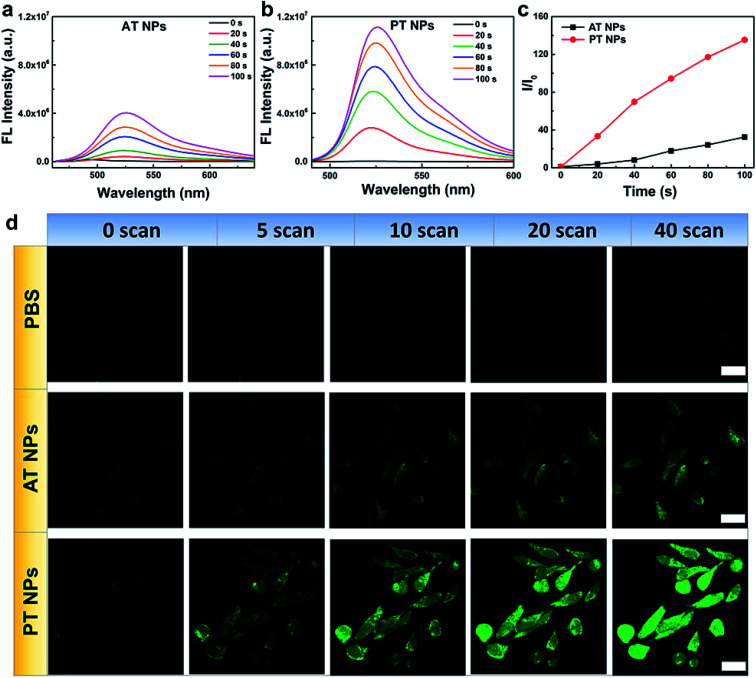
Singlet oxygen generation of TADF NPs. Fluorescence spectra of DCFH in water dispersions of (a) AT NPs and (b) PT NPs after xenon lamp irradiation for different times. (c) Fluorescence intensities of DCFH at the peak wavelength from (a) and (b). (d) Fluorescence images of DCFH-DA in HeLa cells after different treatments and number of scans using a femtosecond laser. Scale bar = 20 μM.

Next, the singlet oxygen generation performance of the TADF NPs was evaluated using dichlorofluorescein (DCFH) as an indicator. In the presence of both AT NPs and PT NPs, the fluorescence intensity of the DCFH increases with prolonged irradiation time under a xenon lamp (one-photon light resource), however, the singlet oxygen generation of PT NPs is much more than that of AT NPs (Fig. 3a–c). Also, the singlet oxygen generation of TADF NPs occurs in a concentration-dependent manner in DCFH solution (Fig. S13a–c†). For a control sample with none of the TADF NPs, DCFH shows no observable emission upon xenon lamp irradiation for 120 s. Moreover, PT NPs show better singlet oxygen generation performance (∼1.9 times) compared to that of AT NPs in HeLa cells under 5 minutes irradiation of the xenon lamp with the same concentration (Fig. S13d and e†). These results are consistent with the aforementioned expectation that smaller Δ*E*_ST_ and *f* are favorable for singlet oxygen generation. To quantitatively evaluate the efficiency of the singlet oxygen generation of TAFD NPs, we measured the ^1^O_2_ quantum yield (*Φ*) using 9,10-anthracenedipropionicacid (ABDA) as an indicator and methylene blue (MB) as a standard reference (Fig. S14–S17†). The ^1^O_2_ quantum yields of the PT and AT NPs were determined to be 13.7 and 7.7% respectively.

The generation of singlet oxygen *via* two-photon excitation of NIR radiation was also studied using DCFH-DA as an indicator. HeLa cells incubated with PBS, AT NPs and PT NPs, respectively were scanned using an 800 nm laser in the confocal microscope (Fig. 3d). For the control sample without any TADF NPs, the green emission of DCFH-DA is very weak even after 40 scans. For the samples incubated with the two TADF NPs, the green emission increases with the number of scans. Again, the brighter fluorescence of DCFH-DA for the PT NP group confirms their better singlet oxygen generation capability compared to AT NPs under two-photon excitation. Fluorescence intensities of DCFH-DA from Fig. 3d indicate that the singlet oxygen generation induced by the two-photon laser of PT NPs is 2.7 times more than that of AT NPs after 40 scans (Fig. S18†). These are attributed to the smaller Δ*E*_ST_ and *f*.

### 2.4 PDT effect of TADF nanoparticles

**Fig. 4:**
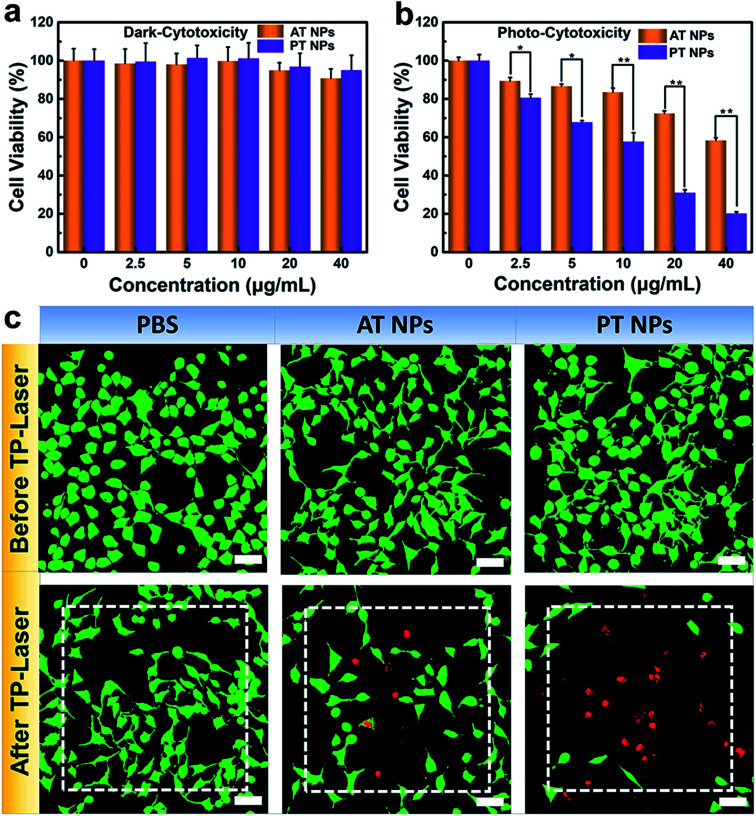
Photo-cytotoxicity of TADF NPs. (a) Biocompatibility (dark-cytotoxicity) and (b) photo-cytotoxicity of PT NPs and AT NPs. (c) Images of HeLa cells stained with Calcein AM/PI after 200 scans using an 800 nm femtosecond laser in the white dash line area. Scale bar = 50 μM. *p < 0.05, **p < 0.01.

To further explore the potential in PDT application, we assessed the biocompatibility of these TADF NPs using MTT assay. There is no significant intrinsic cytotoxicity of TADF NPs without laser irradiation, proving their excellent biosafety (Fig. 4a). Under xenon lamp irradiation, both PT NPs and AT NPs displayed dosage-dependent cytotoxicity towards HeLa cells, illuminating the efficient cytotoxicity of PDT (Fig. 4b). More importantly, the half maximal inhibitory concentration (IC50) of AT NPs (68.2 μg mL^−1^) is 6.5 times higher than that of PT NPs (10.5 μg mL^−1^), indicating that PT NPs achieve more efficient PDT performance than AT NPs. These tests were further repeated with the A549 cell lines and show similar results (Fig. S19†).

A PDT study based on two-photon excitation was carried out on a confocal microscope. To investigate the two-photon PDT effect of TADF NPs in HeLa cells, Calcein-AM (green emission, live cells) and PI (red emission, dead cells) were used to visualize the curative effect. As shown in Fig. 4c, the control group of HeLa cells with or without TADF NP treatments were unable to cause any cell death before two-photon irradiation, however, cells incubated with both TADF NPs showed obvious dead cells (red dots) under two-photon irradiation by a femtosecond laser. Compared with AT NPs, PT NPs show more effective cytotoxicity because more dead cells appear in the irradiation area, fully demonstrating that PT NPs can generate more singlet oxygen than AT NPs under two-photon irradiation and eventually eliminate the cancer cells.

## 3. Conclusion

In conclusion, we have designed and synthesized two TADF molecules AT and PT with AIE characteristics by connecting different D segments with the same A segment. This approach leads to tunable HOMO/LUMO overlaps, and thus, PT achieved a smaller Δ*E*_ST_ of 60 meV and an *f* of 0.03 for enhancing singlet oxygen generation while AT achieved a relatively high Δ*E*_ST_ of 100 meV and *f* of 0.07 for boosting fluorescent emission. Under both one-photon and two-photon excitations, the PT NPs show much stronger singlet oxygen generation capability and PDT performance, while the AT NPs show much brighter fluorescence imaging performance. These results not only suggest that effective PDT and fluorescence imaging can be highly tunable *via* adjusting the Δ*E*_ST_ and *f* of TADF materials but also propose a strategy to design highly efficient PDT and fluorescent imaging agents.

## Author contributions

The manuscript was written through contributions of all authors. All authors have given approval to the final version of the manuscript. Ya-Fang Xiao and Jia-Xiong Chen contributed equally.

## Conflicts of interest

The authors declare no conflict of interest.

## Supplementary Material

SC-011-C9SC05817F-s001
