# Supplementary material for: Manipulating exciton dynamics of thermally activated delayed fluorescence materials for tuning two-photon nanotheranostics
Source: Chem Sci. 2019 Dec 11;11(3):888–95. doi: 10.1039/c9sc05817f (PMC8145712; doi:10.1039/c9sc05817f)
Supplement: SC-011-C9SC05817F-s001 [file SC-011-C9SC05817F-s001.pdf]

Electronic Supplementary Material (ESI) for Chemical Science.
This journal is © The Royal Society of Chemistry 2019

Supplementary Information

Manipulating Exciton Dynamics of Thermally Activated Delayed Fluorescence Materials for Tuning Two-Photon Nanotheranostics

Ya-Fang Xiao, [a] Jia-Xiong Chen, [a,b] Shengliang Li*, [a] Wen-Wen Tao, [b] Shuang Tian, [a] Kai Wang, [b] Xiao Cui, [a] Zhongming Huang, [a] Xiao-Hong Zhang* , [b] and Chun-Sing Lee* [a]

[a] Center of Super-Diamond and Advanced Films (COSDAF) and Department of Chemistry, City University of Hong Kong, Hong Kong SAR, P. R. China.

[b] Jiangsu Key Laboratory for Carbon-Based Functional Materials & Devices, Institute of Functional Nano & Soft Materials (FUNSOM), Joint International Research Laboratory of Carbon-Based Functional Materials and Devices, Soochow University, 199 Ren'ai Road, Suzhou 215123, Jiangsu, P.R. China

Ya-Fang Xiao and Jia-Xiong Chen contributed equally.

Experimental Sections

Materials

Unless stated otherwise, all materials were purchased from commercial sources and used as received. DSPE-PEG2000, Hoechst 33342, 3-(4,5-dimethyl-2-thiazolyl)-2,5-diphenyl-2-H-tetrazolium bromide (MTT), 2',7'-Dichlorodihydrofluorescein diacetate (DCFH-DA), 2',7'-Dichlorodihydrofluorescein (DCFH), 9,10-anthracenedipropionicacid (ABDA), Calcein acetoxymethyl ester (Calcein-AM) and Propidium Iodide (PI) were purchased from Sigma-Aldrich. Dulbecco's Modified Eagle Medium (DMEM), Phosphate-Buffered Saline (PBS, 10X, pH 7.4), Penicillin-Streptomycin (10, 000 U mL^{-1}), Fetal Bovine Serum (FBS), and Trypsin-EDTA (0.5%, no phenol red) were purchased from Thermo Fisher Scientific. Tetrahydrofuran (THF) and Dimethyl Sulfoxide (DMSO) were ordered from J&K Scientific Ltd. Deionized water (DI water) was obtained from a Milli-Q Biocel (Millipore Corporation, Bedford, USA) water purification system. All reagents and solvents were analytical grade and used as received.

Characterization

^{1}H NMR and ^{13}C NMR were recorded on a Bruker 400 spectrometer. Mass spectra data were achieved via Trace-ISQ gas chromatography-mass spectrometer and Applied Biosystems API-2000 liquid chromatography/mass spectrometry/mass spectrometry (LC/MS/MS) Triple-Q mass spectrometer. Density functional theory (DFT) calculations were performed using Gaussian 09, and molecular structures at ground

state were optimized at the B3LYP/6-31G(d) level. Fluorescence spectra were measured using a Spectrofluorometer Fluormax-4 (HORIBA Jobin Yvon Inc.). UV-Vis absorption spectra were recorded using an Ultra-Violet Visible Scanning Spectrophotometer Shimadzu 1700. Photoluminescence quantum yields were measured using an integrating sphere under air conditions, and transient PL measurements were measured with an Edinburgh Instruments FLS980 spectrometer under oxygen-free conditions. Transmission electron microscopy (TEM) images were observed using a FEI / Philips Tecnai 12 BioTWIN. DLS analysis measurements were recorded using a Dynamic Light Scattering Particle Size Analyzer (Malvern Zetasizer Nano ZS). One-photon fluorescence images of the cells were taken using a Laser Confocal Scanning Microscope (Leica SPE). Two-photon fluorescence images of the cells were taken using another Laser Confocal Scanning Microscope (Leica TCS SP5). All photos were taken by a digital SRL camera.

Synthesis

N-(2-chlorophenyl)-10-phenyl-10H-phenoxazin-3-amine (1)

2-chloroaniline (6 mmol), 3-bromo-10-phenyl-10H-phenoxazine (5 mmol), Cs_2CO_3 (10 mmol), $P(t\text{-}Bu)_3$ (0.6 mmol) and $PdOAc_2$ (0.2 mmol) are dissolved in 100 mL of toluene. Then, the reaction solution is stirred to reflux for 12 h. After completion of the reaction, water and dichloromethane (DCM) are added. The organic layer is concentrated in vacuo and the residue solid is purified to give the product as white solid (1.51g, 79%). ^1H NMR (400 MHz, Acetone-d6) δ 7.74 (t, J = 7.4 Hz, 2H), 7.60 (t, J =

7.5 Hz, 1H), 7.47 (d, J = 7.5 Hz, 2H), 7.38 (d, J = 7.9 Hz, 1H), 7.23 - 7.15 (m, 2H), 6.89 - 6.55 (m, 6H), 5.95 (s, 2H). MS (EI) m/z: $[M]^+$ calcd for $C_{24}H_{17}ClN_2O$ 384.10, found 384.31.

12-phenyl-5,12-dihydroindolo[3,2-b]phenoxazine (P)

1 (2 mmol), 1,3-bis(2,6-di-i-propylphenyl)imidazolium chloride (0.1 mmol), $PdOAc_2$ (0.3 mmol), K_2CO_3 (4 mmol) and 40 mL of N,N-dimethylacetamide (DMF) are added into a 100 mL round-bottom flask. Then, the mixture is stirred at 130 °C for one day. Then, water and DCM are added to the cooled mixture. The organic layer is concentrated and the residue solid is purified to give the product as white solid (0.42g, 60%). ^1H NMR (400 MHz, Acetone-d6) δ 10.07 (s, 1H), 7.75 (dt, J = 10.0, 1.9 Hz, 2H), 7.61 (ddd, J = 8.0, 3.4, 2.2 Hz, 2H), 7.52 - 7.48 (m, 2H), 7.37 (d, J = 8.1 Hz, 1H), 7.24 - 7.19 (m, 1H), 7.01 – 6.96 (m, 1H), 6.93 (s, 1H), 6.76 (dt, J = 6.4, 3.6 Hz, 1H), 6.69 - 6.64 (m, 2H), 6.59 (s, 1H), 5.98 - 5.94 (m, 1H). MS (EI) m/z: $[M]^+$ calcd for $C_{24}H_{16}N_2O$ 348.13, found 348.11.

2-(12-phenylindolo[3,2-b]phenoxazin-5(12H)-yl)thianthrene 5,5,10,10-tetraoxide (PT)

P (1.2 mmol), 2-bromothianthrene 5,5,10,10-tetraoxide (1 mmol), Cs_2CO_3 (2 mmol), $P(t\text{-}Bu)_3$ (0.15 mmol) and $PdOAc_2$ (0.05 mmol) are dissolved into toluene solution. The mixture is then reflux overnight. After that, DCM and water are added to the cooled mixture, and gave the residue solid, then purified by column chromatography to give the product as red solid (0.42g, 67%). ^1H NMR (400 MHz, $CDCl_3$) δ 8.45 (dd, J = 7.1, 5.2 Hz, 2H), 8.34 - 8.30 (m, 2H), 7.98 (dd, J = 8.3, 2.1 Hz, 1H), 7.90 - 7.86 (m, 2H), 7.70 - 7.64 (m, 3H), 7.56 (t, J = 7.5 Hz, 1H), 7.43 (d, J = 7.4 Hz, 2H), 7.39 (d, J = 8.2

Hz, 1H), 7.29 (t, J = 7.6 Hz, 1H), 7.17 (t, J = 7.5 Hz, 1H), 6.86 (s, 1H), 6.73 - 6.71 (m, 1H), 6.67 - 6.61 (m, 2H), 6.51 (s, 1H), 5.96 (d, J = 7.6 Hz, 1H). ^{13}C NMR (101 MHz, $CDCl_3$) δ 143.53, 143.23, 141.51, 139.65, 139.33, 139.24, 135.94, 134.43, 133.93, 133.73, 131.33, 130.85, 129.75, 128.72, 128.23, 126.26, 126.02, 125.57, 124.39, 123.52, 122.68, 121.63, 121.02, 119.90, 119.30, 115.43, 113.20, 109.24, 103.92, 97.22. MS (EI) m/z: $[M]^+$ calcd for $C_{36}H_{22}N_2O_5S_2$ 626.10, found 626.30. Anal. calcd for $C_{36}H_{22}N_2O_5S_2$ C, 69.00; H, 3.54; N, 4.47; O, 12.76; S, 10.23; found C, 68.73; H, 3.66; N, 4.82; O, 12.34; S, 10.45.

2-(13,13-dimethyl-5-pheny5,13-dihydro-11H-indolo[2,3-b]acridin-11-yl)thianthrene 5,5,10,10-tetraoxide (AT)

AT is synthesized using the similar procedure as for PT. With that, P is replace by 13,13-dimethyl-5-phenyl-11,13-dihydro-5H-indolo[2,3-b]acridine (A) (0.41g, 64%). ^1H NMR (400 MHz, CD_2Cl_2) δ 8.53 (d, J = 2.1 Hz, 1H), 8.48 (d, J = 8.4 Hz, 1H), 8.34 – 8.29 (m, 2H), 8.09 (dd, J = 8.4, 2.1 Hz, 1H), 7.93 - 7.88 (m, 2H), 7.80 (d, J = 7.7 Hz, 1H), 7.74 (t, J = 7.6 Hz, 2H), 7.66 - 7.61 (m, 2H), 7.48 (dd, J = 11.6, 7.9 Hz, 4H), 7.40 - 7.36 (m, 1H), 7.23 (d, J = 7.5 Hz, 1H), 6.99 (s, 3H), 6.35 (s, 1H), 1.71 (s, 6H). ^{13}C NMR (101 MHz, CD_2Cl_2) δ 143.65, 141.49, 139.88, 139.57, 139.31, 137.69, 135.62, 134.83, 134.12, 133.95, 131.59, 131.30, 131.05, 129.97, 129.60, 128.40, 128.34, 126.37, 126.19, 125.93, 124.68, 124.36, 122.83, 121.29, 120.38, 116.92, 114.04, 109.32, 105.62, 104.86, 36.99, 30.16. MS (EI) m/z: $[M]^+$ calcd for $C_{39}H_{28}N_2O_4S_2$ 652.15, found 652.40. Anal. calcd for $C_{39}H_{28}N_2O_4S_2$ C, 71.76; H, 4.32; N, 4.29; O, 9.80; S, 9.82; found C, 72.06; H, 4.55; N, 4.31; O, 9.69; S, 9.39.

Preparation of AT NPs and PT NPs

250 μL of AT /THF (0.5 mg/mL) and 250 μL of DSPE-PEG2000/THF (5 mg/mL) were fully mixed and then added dropwise into 5 mL of DI water under 1200 rpm magnetic stirring at 25°C for 1 h. Then THF was removed from the original AT NPs suspensions using a rotary evaporator. The PT NPs were prepared by the same protocol. The AT NPs and PT NPs suspensions were stored at 4 °C for future use.

Cell culture

HeLa cells and A549 cells were obtained from American Type Culture Collection (ATCC, Manassas, VA, USA) and cultured in Dulbecco's Modified Eagle's medium (DMEM). The culture medium contains 10% of fetal bovine serum (FBS) and antibiotics (penicillin and streptomycin) of 50 units per milliliter at 37 °C under a humidified atmosphere containing 5% CO_2.

One-photon and two-photon fluorescence imaging of HeLa cells

HeLa cells (1×10^4) were seeded into 35 mm microscopy dishes with a glass bottom and incubated at 37°C overnight. Before observation, HeLa cells were incubated with AT NPs (PT NPs) with the final concertation of 40 μg/mL for 8 h. The one-photon and two-photon fluorescence of AT NPs (PT NPs) were observed by a Confocal Laser Scanning Microscope (CLSM) with a femtosecond laser. The excitation laser source for one-photon fluorescence is 405 nm and that for two-photon fluorescence is 800 nm. The emission wavelength range is 550-700 nm. The fluorescence intensities of AT NPs

(PT NPs) in cells were calculated by the CLSM software. For the co-localization with nuclei, Hoechst 33342 was used to stain the nuclei before CLSM observation.

$^1O_2$ quantum yield (Φ) measurements

Quantum yields of $^1O_2$ induced by TADF NPs were measured by using ABDA as a sensor under irradiation with a xenon lamp. The absorption of ABDA decreases proportionally with increased $^1O_2$ in the water phase. Methylene blue was used as a calibration standard (Φ = 52%). The decreased absorption of ABDA was monitored by an ultra-violet and visible spectrophotometer. All samples were irradiated with a xenon lamp of 100 mW cm^{-2}. The absorption maxima of methylene blue and the TADF NPs were adjusted to ~ 0.15-0.25 OD. $^1O_2$ quantum yield of the TADF NP samples were calculated using the following formula:

$$\Phi_{sample} = \Phi_{MB}\left(\frac{K_{sample}}{K_{MB}}\right)\left(\frac{F_{MB}}{F_{sample}}\right) \qquad (1)$$

where K_{sample} and K_{MB} are the decrease rate constants of the ABDA by the TADF NPs and methylene blue at 399 nm, respectively.

$$F_{MB/sample} = 1 - 10^{-A_{MB/sample}} \qquad (2)$$

A_{MB} and A_{sample} represent the absorption integral of the methylene blue and TADF NPs in the wavelength range 400-700 nm, respectively.

Two-photon excited (TPE) emission spectra measurement

Two-photon excited emission spectra of AT NPs and PT NPs were measured by a fluorospectrophotometer with a femtosecond pulse laser. The excitation source was from an optical parametric amplifier (TOPAS-C) pumped by a mode-locked Ti: sapphire laser oscillator/amplifier system (Spectra Physics). The pulse width of the laser was ~ 120 fs and the repetition rate was 1 kHz. The fluorescence was vertically collected into an Acton monochromator system by a telescope system. The monochromator connected with a GaAs photomultiplier tube (Hamamatsu) was used as the recorder for the two-photon induced up-converted fluorescence.

Detection of ROS generation in vitro and in vivo

For in vitro ROS detection, the AT NPs (PT NPs) were added into DCFH/PBS solution with the final concertation of 2.5, 5, 10 μg/mL. Then, all the samples were irradiated by a xenon lamp under 100 mW/cm^2 for 120 s. The fluorescence intensity of DCFH was measured by the spectrofluorometer to investigate the ROS generation induced by one-photon laser. For in vivo ROS detection, HeLa cells were seeded into 35 mm microscopy dishes with glass bottom and treated with AT NPs (PT NPs) for 8 h. Then, all the cells were stained by the DCFH-DA/PBS solution for 30 min at 37 ℃ and then irradiated by the xenon lamp (one-photon excitation) under 100 mW/cm^2 for 120 s. The fluorescence of DCFH-DA was observed by the CLSM and the fluorescence intensity of DCFH-DA in cells was calculated by the CLSM software. For the detection of ROS generation induced by two-photon laser, cells were scanned with different numbers by the femtosecond laser source (800 nm) installed at the CLSM platform after stained

with the DCFH-DA/PBS. The fluorescence of DCFH-DA was observed by CLSM after different scan numbers and the fluorescence intensity of DCFH-DA in cells was calculated by the CLSM software.

Cytotoxicity assay

The cytotoxicity experiments were carried out using the MTT assay. HeLa cells (or A549 cells, ~ 5000 cells/well) were seeded on a 96-well plate in 100 μL of complete DMEM for 12 h incubation. Then, the AT NPs (PT NPs) were added into cells with different final concentrations (2.5, 5, 10, 20 and 40 μg/mL). After 8 h incubation, cells were irradiated under xenon Lamp (200 mW/cm^2) for 5 min. After another 16 h incubation, the cell viabilities were measured by MTT assay. To evaluate the dark cytotoxicity of AT NPs (PT NPs), the same concentrations of nanoparticles were added into cells in 96-well plate for 24 h in darkness and then the cell viabilities were measured. Cells without NPs were set as a control for 100% viability.

Calcein AM/PI Assay for two-photon induced PDT

HeLa cells (~1×10^5/plate) were seeded in 35 mm microscopy dishes with glass bottom and cultured for 12 h. Then, the cells were incubated with AT NPs (PT NPs) for 8 h and then scanned with or without two-photon laser (femtosecond laser source: 800 nm) for 200 cycles. After another 4 h incubation, the cells were stained with Calcein AM/PI for 30 min and then washed twice with PBS. The excitations of Calcein AM and PI is 488 nm and 514 nm, respectively. Finally, all the groups of cells were imaged by the CLSM.

Statistical analysis

Error bars were based on the standard error of the mean (SEM) and n = 3. The data are given as Mean ± SEM. Statistical significance was analyzed by the two-tailed Student's t test. *$p < 0.05$ and **$p < 0.01$ was considered statistically significant.

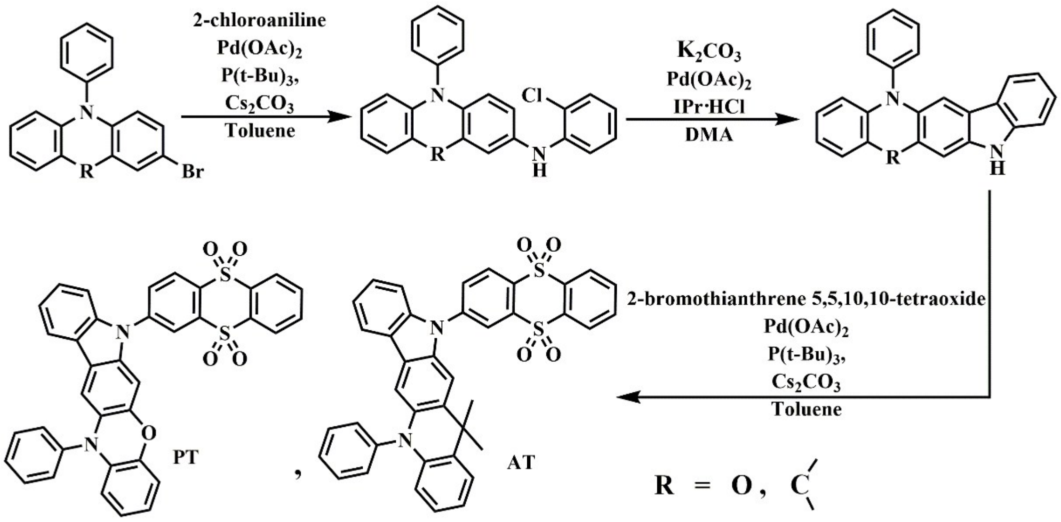

Scheme S1. Synthetic routes of PT and AT.

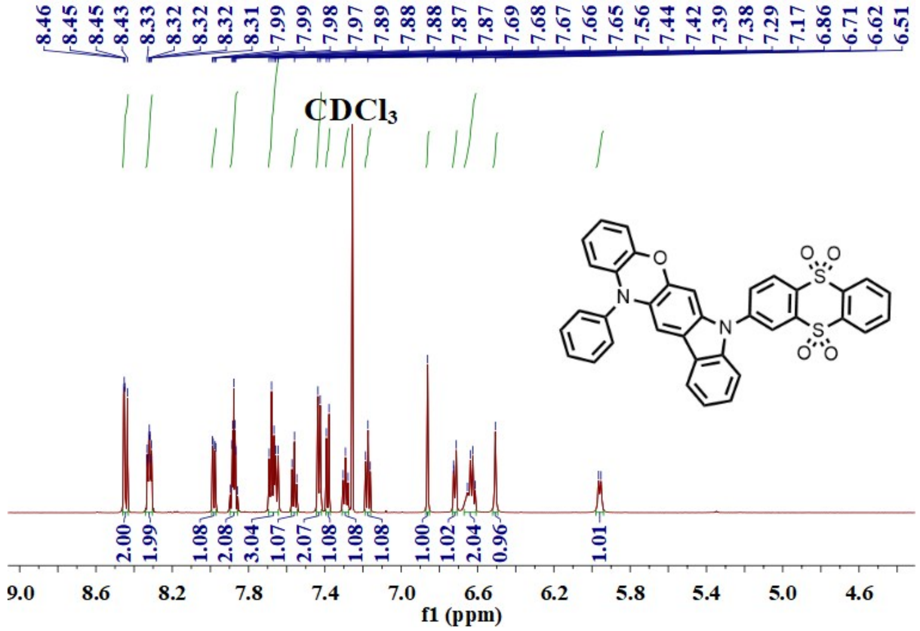

Figure S1. ^{1}H NMR spectrum of PT in CD_2Cl_2.

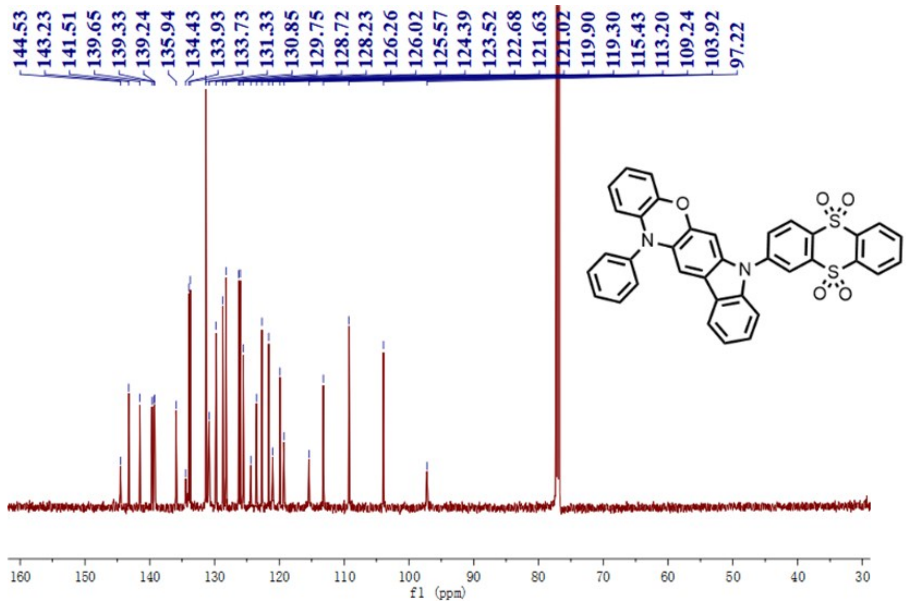

Figure S2. ^{13}C NMR spectrum of PT in acetone.

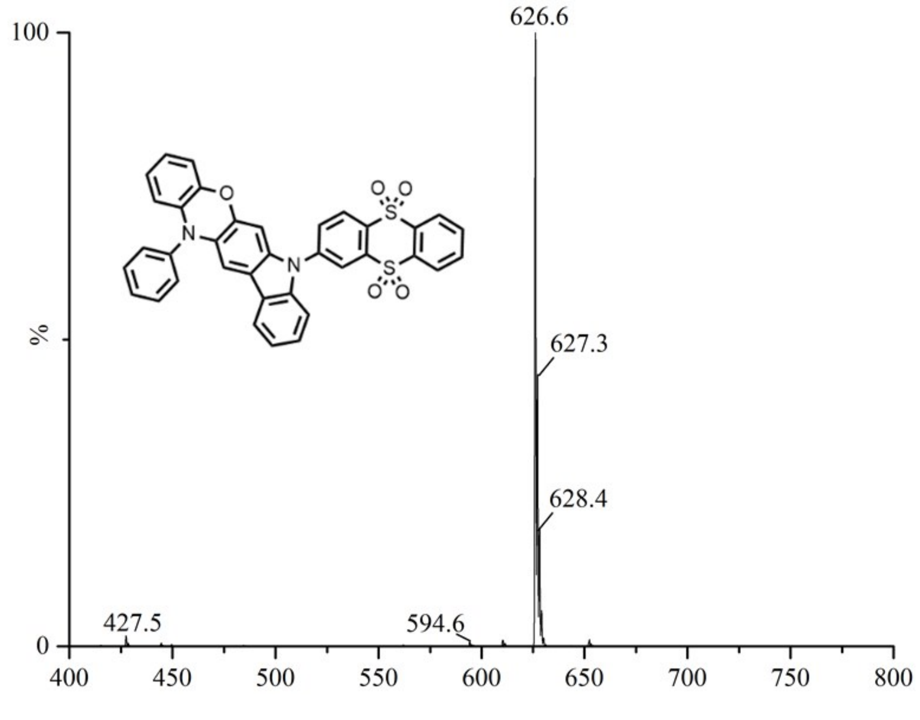

Figure S3. High-resolution mass spectrum of PT.

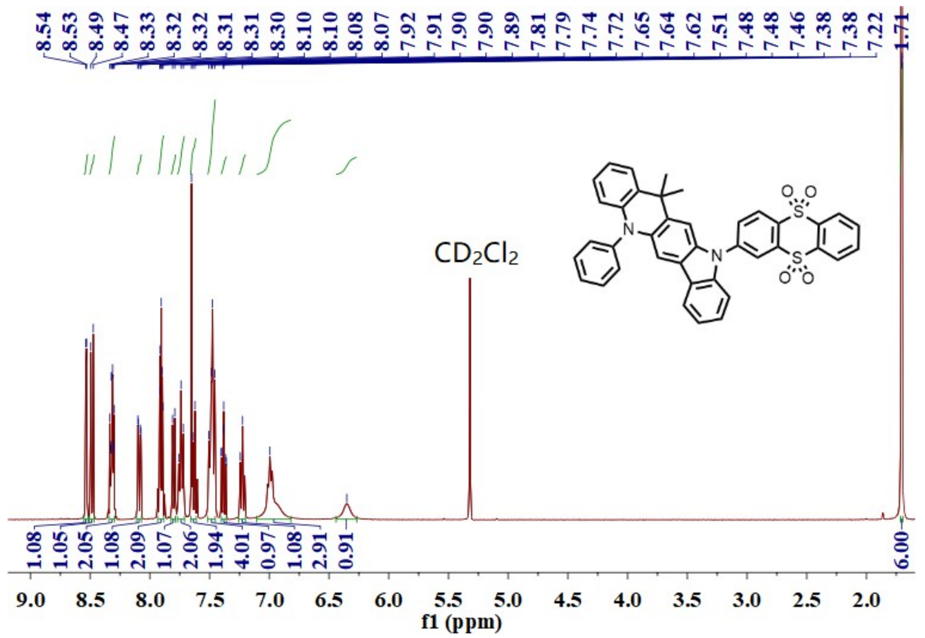

Figure S4. ^{1}H NMR spectrum of AT in CD_2Cl_2.

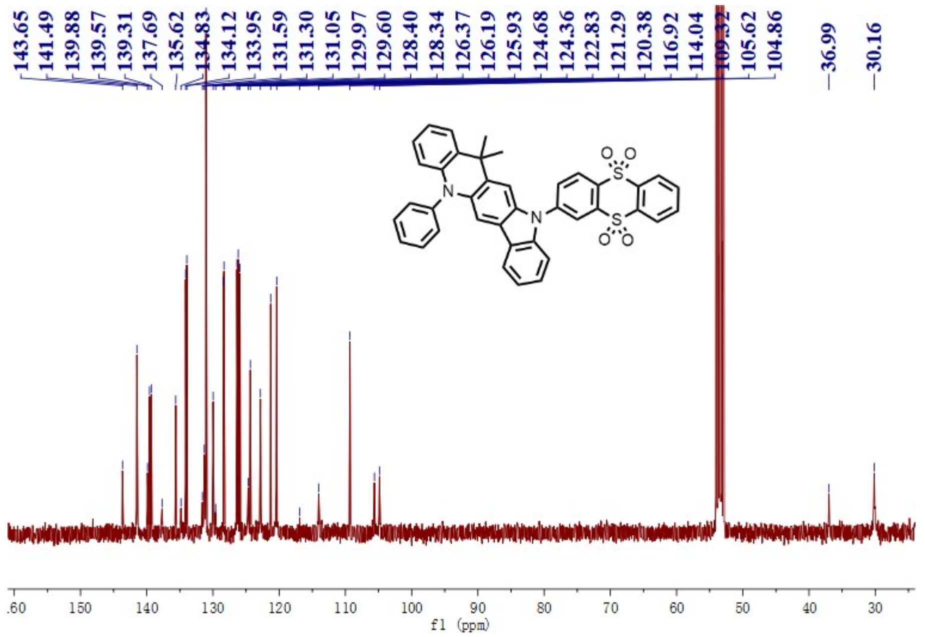

Figure S5. ^{13}C NMR spectrum of AT in acetone.

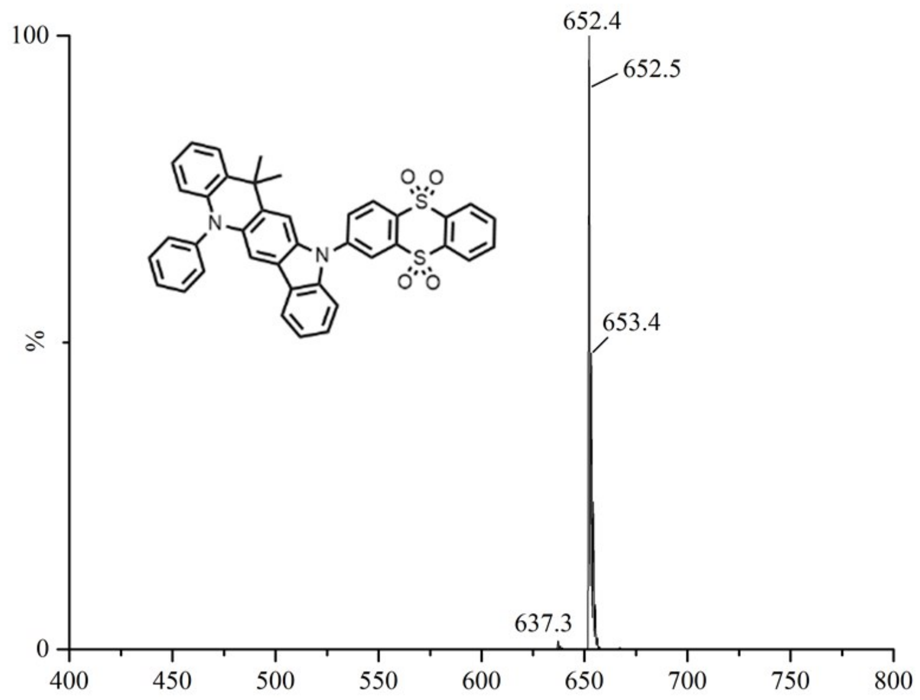

Figure S6. High-resolution mass spectrum of AT.

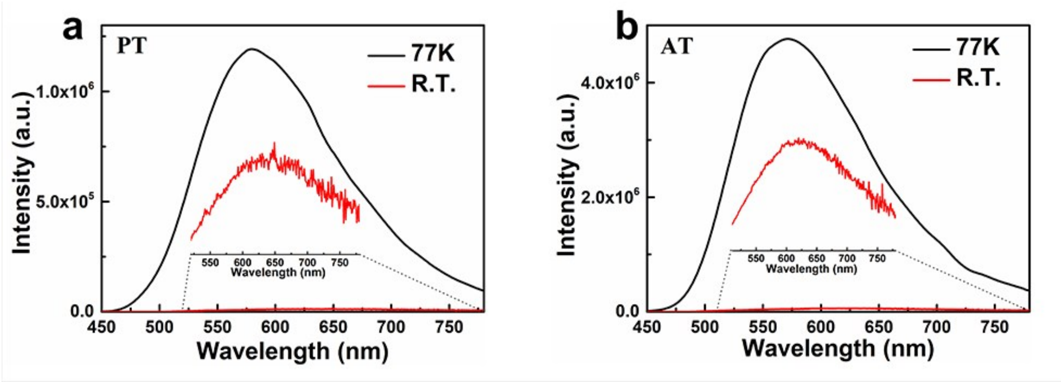

Figure S7. Fluorescence spectra of PT (a) and AT (b) in 2Me-THF solution measured at room temperature and 77 K.

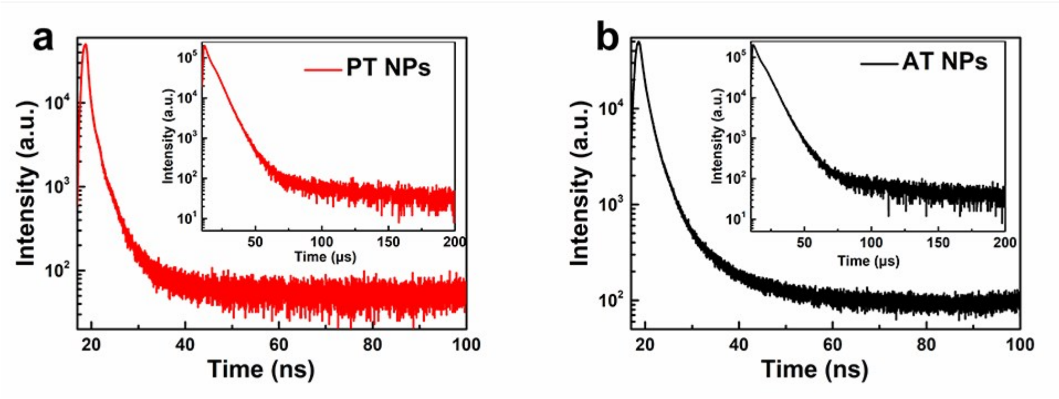

Figure S8. Prompt transient PL decay curves and delayed transient PL decay curves (inset photo) at room temperature of (a) PT NPs and (b) AT NPs.

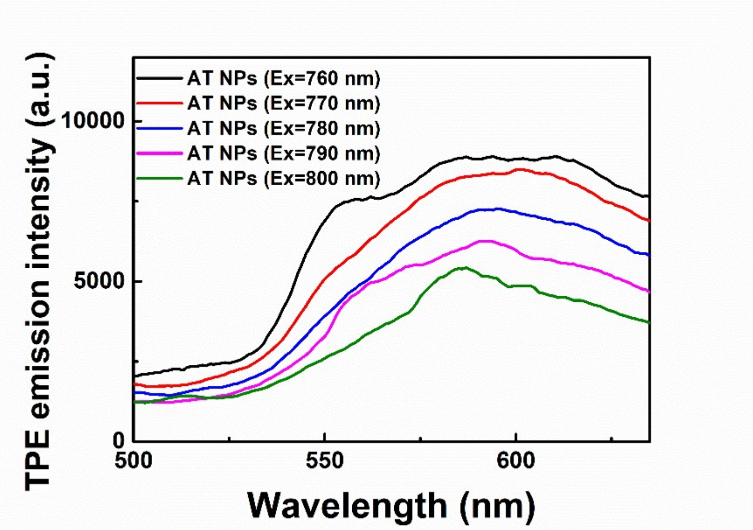

Figure S9 The TPE emission spectra of AT NPs (0.1 mg/mL) under 760, 770, 780, 790, 800 nm femtosecond laser excitation.

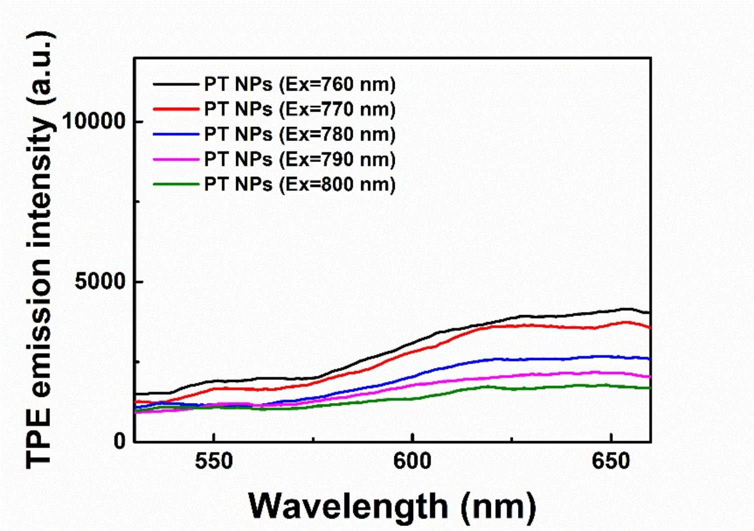

Figure S10. The TPE emission spectra of PT NPs (0.1 mg/mL) under 760, 770, 780, 790, 800 nm femtosecond laser excitation.

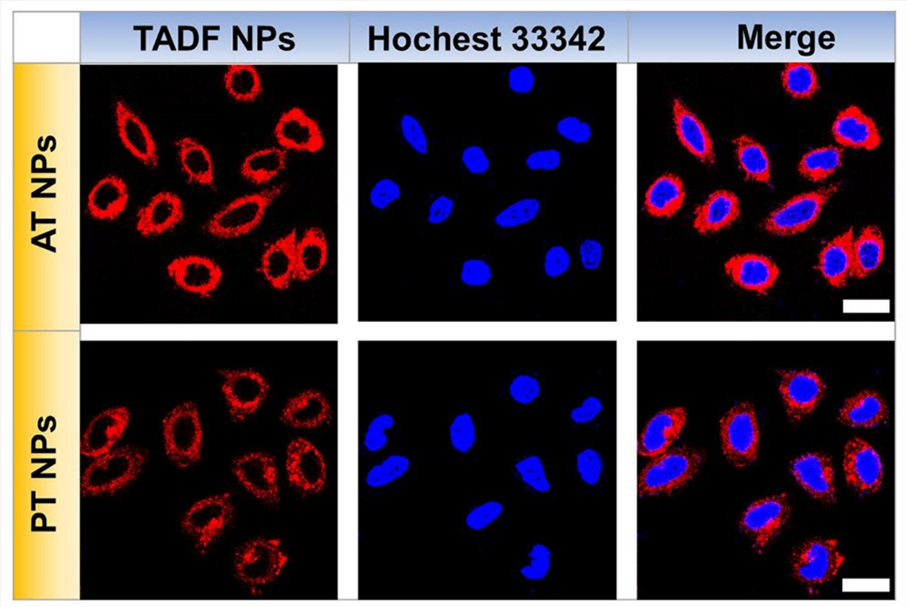

Figure S11. Fluorescent images of AT NPs and PT NPs in HeLa cells (excitation: 405 nm, emission: 550-700 nm). The nuclei were stained by Hoechst 33342 (excitation: 405 nm, emission: 430-450 nm). Scale bar = 20 μM.

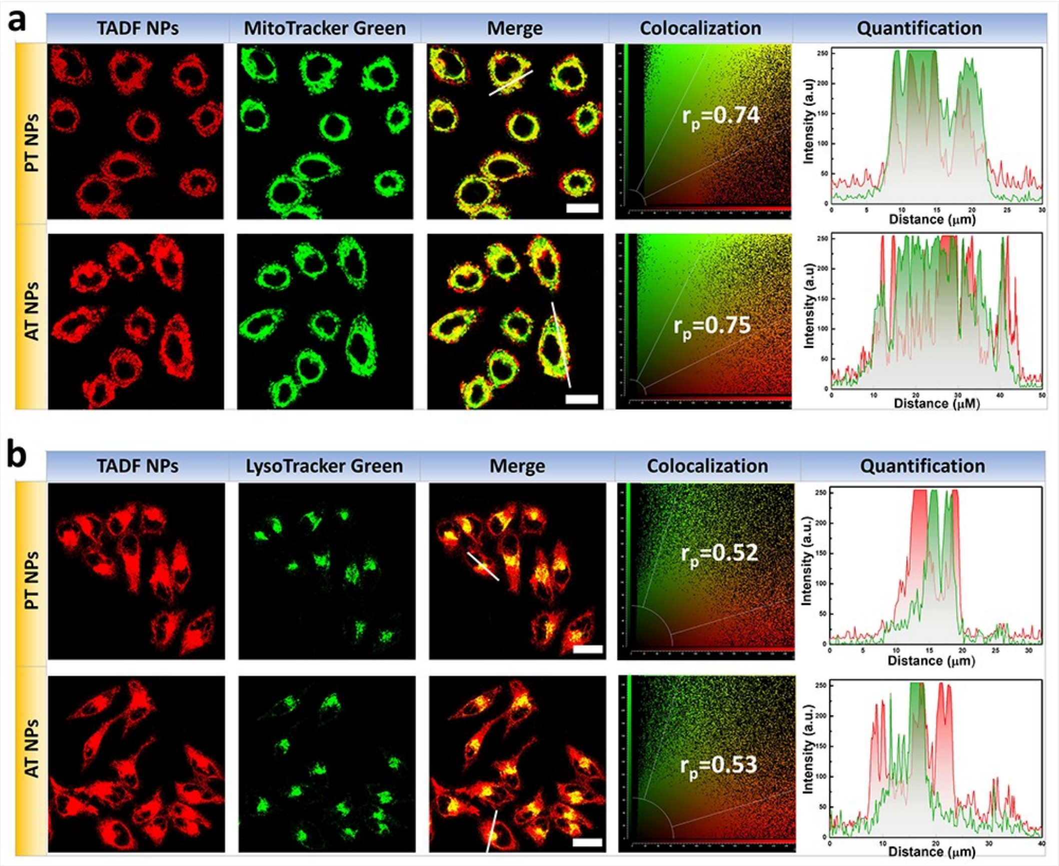

Figure S12. (a) Representative mitochondria co-localization images of the TADF NPs by CLSM in HeLa cells, where r_p indicates Pearson's co-localization coefficient; (b) Representative lysosome co-localization images of the TADF NPs by CLSM in HeLa cells, where r_p indicates Pearson's co-localization coefficient. Fluorescence topographic profiles display fluorescence intensity along the white lines marked in TADF NPs channel (red) and organelle-tracker channel (green), respectively. Scale bar = 20 μM.

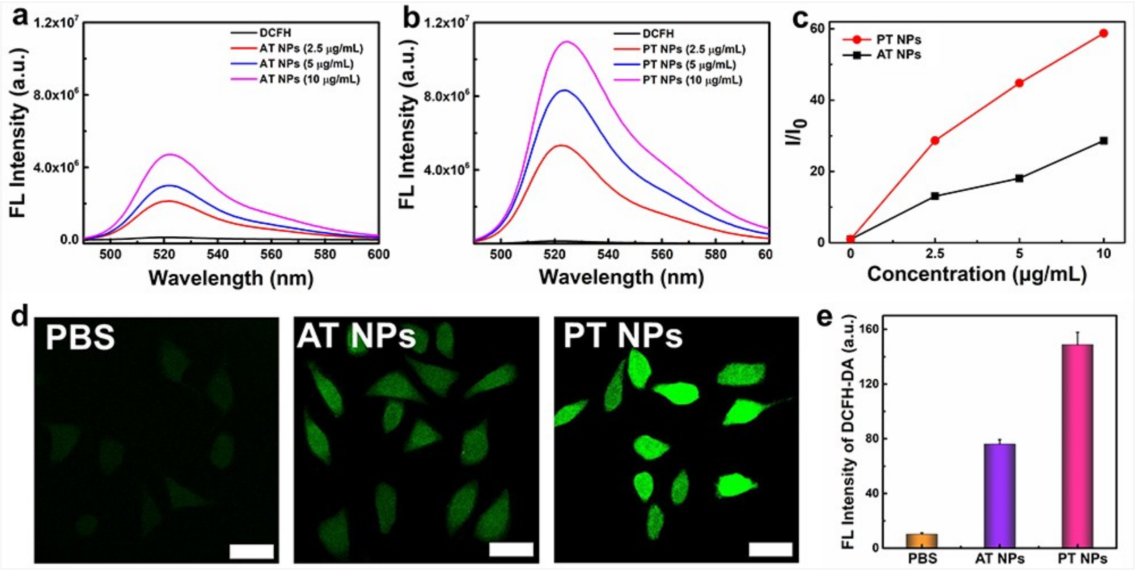

Figure S13. Fluorescent spectra of DCFH in (a) AT NPs and (b) PT NPs solutions with different concentrations under the one-photon laser for 120 s. (c) Quantitative fluorescence values of DCFH at 523 nm in (a,b). (d) Fluorescent images of DCFH-DA in HeLa cells after incubation of PT NPs, AT NPs and PBS for 8 h under one-photon irradiation (xenon lamp, 5 min). (e) Quantitative fluorescence values of DCFH-DA in HeLa cells from (d). Fluorescence calculation was conducted by the CLSM software.

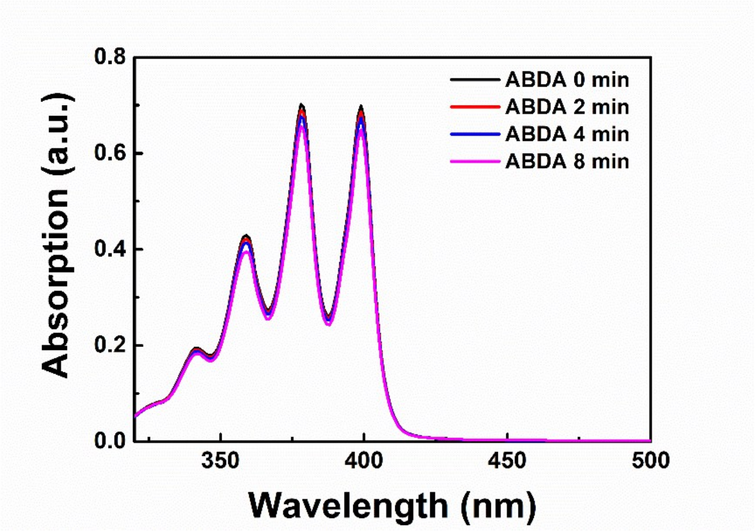

Figure S14. Time-dependent bleaching of ABDA caused by $^1O_2$ generated without any other chemicals under a xenon lamp.

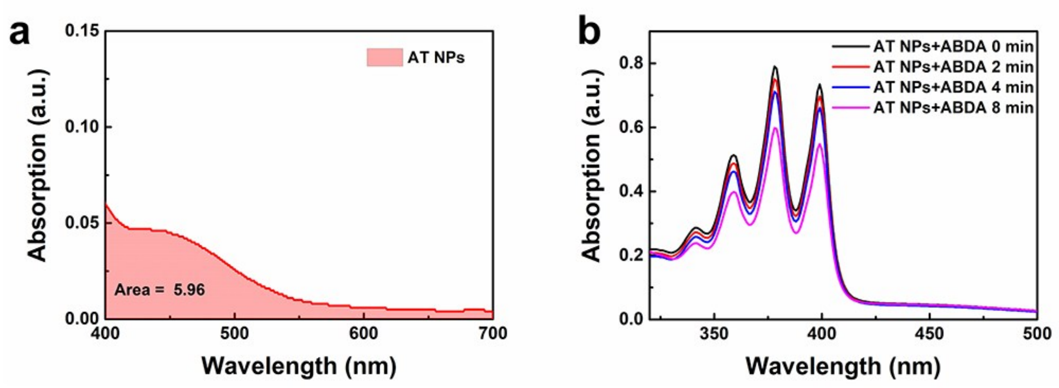

Figure S15. (a) The absorption peak area of AT NPs dispersed in deionized water. (b) Time-dependent bleaching of ABDA caused by $^1O_2$ generated by AT NPs under a xenon lamp.

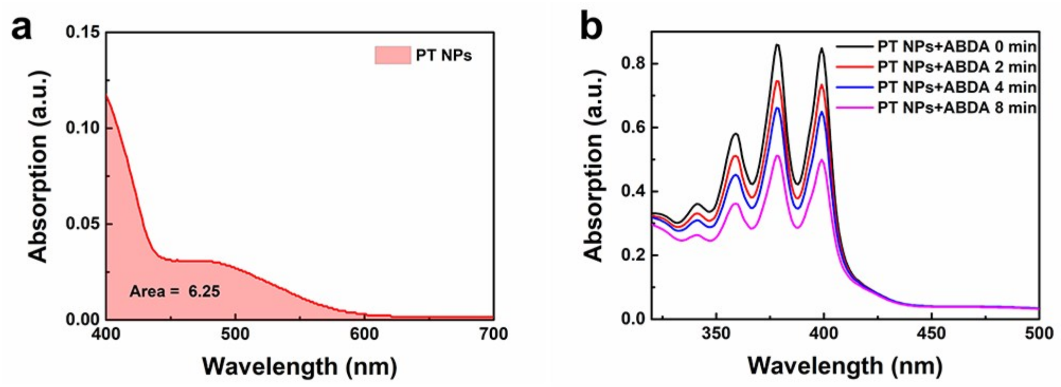

Figure S16. (a) The absorption peak area of PT NPs dispersed in deionized water. (b) Time-dependent bleaching of ABDA caused by $^1O_2$ generated by PT NPs under a xenon lamp.

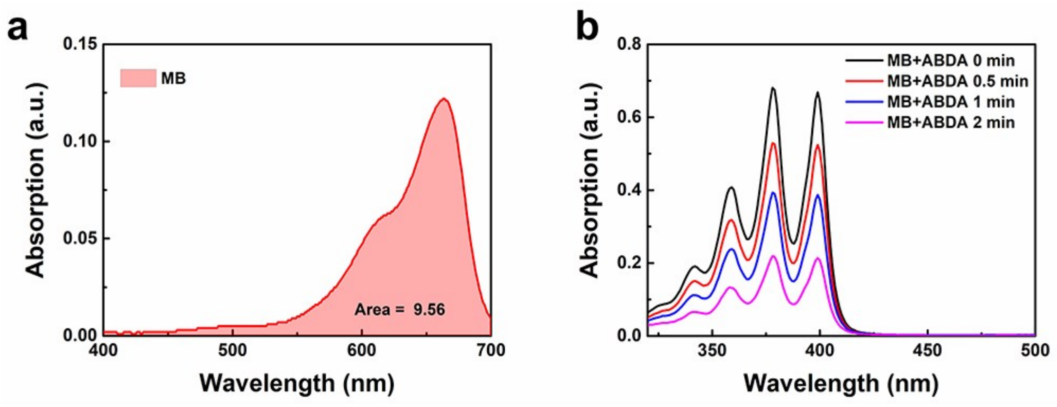

Figure S17. (a) The absorption peak area of MB dispersed in deionized water. (b) Time-dependent bleaching of ABDA caused by $^1O_2$ generated by MB under a xenon lamp.

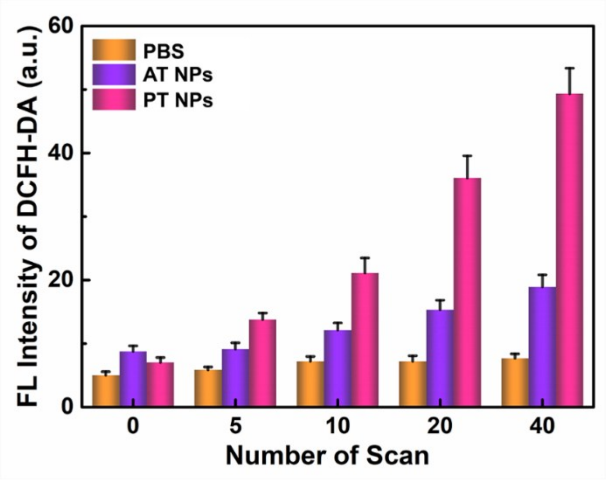

Figure S18. Quantitative fluorescence values of DCFH-DA in HeLa cells from Figure 3d.

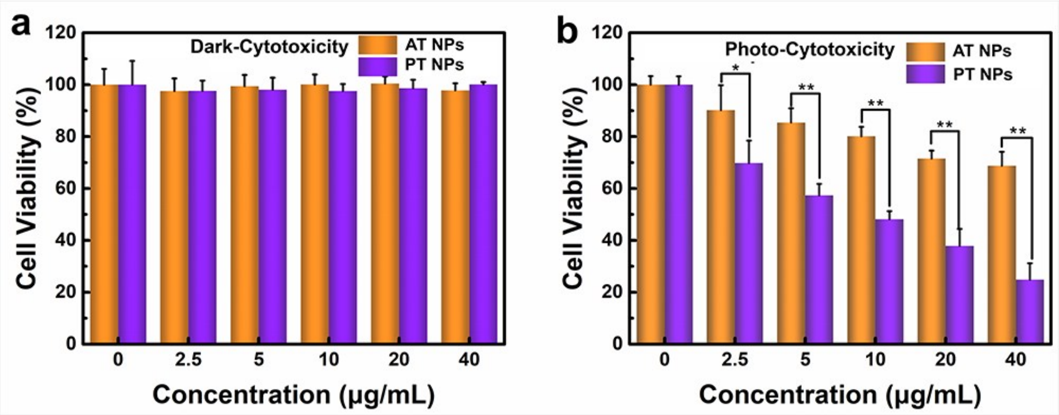

Figure S19. (a) Dark-cytotoxicity of PT NPs and AT NPs without one-photon laser for 24 h with A549 cell line. (b) Photo-cytotoxicity of PT NPs and AT NPs under the one-photon laser irradiation with A549 cell line. *p < 0.05, **p < 0.01
